# Co-Design to Support the Development of Inclusive eHealth Tools for Caregivers of Functionally Dependent Older Persons: Social Justice Design

**DOI:** 10.2196/18399

**Published:** 2020-11-09

**Authors:** Karine Latulippe, Christine Hamel, Dominique Giroux

**Affiliations:** 1 Department of Studies of Teaching and Learning Laval University Québec, QC Canada; 2 Département de réadaptation Faculté de médecine Laval University Québec, QC Canada; 3 Centre d'Excellence du Vieillissement de Québec Chu de Québec Québec, QC Canada

**Keywords:** caregivers, aged, help-seeking behavior, community-based participatory research, eHealth, telemedicine, health care disparities

## Abstract

**Background:**

eHealth can help reduce social health inequalities (SHIs) as much as it can exacerbate them. Taking a co-design approach to the development of eHealth tools has the potential to ensure that these tools are inclusive. Although the importance of involving future users in the development of eHealth tools to reduce SHIs is highlighted in the scientific literature, the challenges associated with their participation question the benefits of this involvement as co-designers in a real-world context.

**Objective:**

On the basis of Amartya Sen’s theoretical framework of social justice, the aim of this study is to explore how co-design can support the development of an inclusive eHealth tool for caregivers of functionally dependent older persons.

**Methods:**

This study is based on a social justice design and participant observation as part of a large-scale research project funded by the Ministry of Families as part of the Age-Friendly Quebec Program (Québec Ami des Aînés). The analysis was based on the method developed by Miles and Huberman and on Paillé’s analytical questioning method.

**Results:**

A total of 78 people participated in 11 co-design sessions in 11 Quebec regions. A total of 24 preparatory meetings and 11 debriefing sessions were required to complete this process. Co-designers participated in the creation of a prototype to support the search for formal services for caregivers. The majority of participants (except for 2) significantly contributed to the tool’s designing. They also incorporated conversion factors to ensure the inclusiveness of the eHealth tool, such as an adequate level of digital literacy and respect for the caregiver’s help-seeking process. In the course of the experiment, the research team’s position regarding its role in co-design evolved from a neutral posture and promoting co-designer participation to one that was more pragmatic.

**Conclusions:**

The use of co-design involving participants at risk of SHIs does not guarantee innovation, but it does guarantee that the tool developed will comply with their process of help-seeking and their literacy level. Time issues interfere with efforts to carry out a democratic process in its ideal form. It would be useful to single out some key issues to guide researchers on what should be addressed in co-design discussions and what can be left out to make optimal use of this approach in a real-world context.

## Introduction

### Background

The growing emergence of eHealth tools in recent years clearly demonstrates the interest that both individuals and health institutions take in them [1]. Since the onset of the COVID-19 pandemic, this interest has increased. eHealth—the digital tools designed to improve health—enables people to access health information anytime, anywhere [2] and holds the promise of improving access to health care and services [3-6]. However, a literature review has brought to light a digital divide, that is, the unequal opportunity to use eHealth among population groups, which further exacerbates the existing social health inequalities (SHIs) [7]. SHIs are differences between population groups in their health status, including life expectancy and comorbidities, because of modifiable social factors [8]. Already present in our communities, SHI is accentuated by eHealth because the people who have difficulty using eHealth tools are the same people who are at risk of SHIs [7]. Nevertheless, promising solutions have been identified to reduce the risk that eHealth may exacerbate SHI and reap its benefits instead [7]. One of these solutions is to involve people at risk of SHIs in the development of eHealth tools [9,10].

### Participatory Research and Co-Design

This participation can be achieved in several ways. Participatory research can be broken down to more specific approaches, including community-based participatory research, action research, participative evaluation, emancipatory evaluation, and co-design. According to the various studies that claim to be participatory, the roles that future users (FUs) play vary from consultant to research team member [11,12]. However, in participatory research, there is an expectation of research co-governance by academic researchers and the target population (eg, FUs of knowledge, services, innovation, research findings, etc) [13]. In research design, and in the health field in particular, there is an increasing amount of research on co-creation or co-design of services, programs, or tools together with FUs [14]. Co-design implies that FUs are considered co-designers in the same way as the research team [15]. This involves cooperation between various experts, such as researchers, designers, or developers, and FUs (experts in their own experience). Particular attention is paid to the participation of FUs in the design process and the centralization of their experiences [16]. It is important to note that FUs are involved in all stages of the creative process and that they are part of the team. This is different from other processes where there is a back and forth between the research team that creates the tool and the collection of data from FUs (interviews or observations) [15]. For the purpose of comparison, interviewing FUs allows designers to listen to and interpret what others *say* and observation focuses on seeing what others are *doing* and how they use products or services, whereas co-design enables FUs to jointly *explore* and *create* solutions [16,17].

### Arguments in Favor of Using Participatory Approaches

Researchers and designers advocating participatory approaches such as co-design argue, in particular, that the involvement of FUs in the development of a digital tool makes it possible to determine the interests and capacities of FUs and their attitudes, beliefs, values, and expectations [10,18-20], which increases the chances of developing a universally accessible tool [10]. Universal access includes equipment, internet connections, skills development, ongoing technical support, and appropriate content [10], thereby precluding SHI exacerbation through eHealth. Just as patient partners support the relevance and utility of ongoing research, digital patients (e-patients) can contribute to the development of an equally useful and relevant eHealth tool [21]. Involving patients or FUs in research and innovation also helps to redirect and improve the research project, reduce clinical uncertainty, and accelerate the adoption of significant and highly promising results, with the ultimate goal of improving the health care experience and health outcomes [22,23]. In addition, it is possible that FU participation may prove to be a beneficial element in service design, leading to innovation [24]. Finally, the participation of FUs promotes mutual understanding of the context in which the eHealth tool is used [25]. Advocates of participatory and co-design research stress the fact that these approaches increase the cultural and logistical relevance of action in the community, support empowerment, and promote knowledge transfer [26]. It is also argued that these approaches highlight facilitators and social, political, and economic barriers to the knowledge and resources needed for health [13]. At the individual level, people who engage in participatory research processes perceive beneficial effects on their physical and psychological health, self-confidence, self-esteem, empowerment, and social relationships [27]. Using traditional data collection methods such as interviews and observation to grasp multiple facets of the FU’s situation would require asking the right questions, conducting the interview or observation at the right time, and interpreting findings in the right way. Co-design enables co-designers to incorporate all this information into the development of the tool [15]. All these arguments support the idea that co-design can contribute to the reduction of SHIs through the opportunity to make the developed tool relevant and accessible to all FUs.

### Arguments That Add Nuance to the Use of Participatory Approaches

Researchers and designers have raised important counter-arguments to nuance the potential benefits of co-design. Among other things, people may not be aware of their needs, may be unable to express their needs, or may be unwilling to discuss them in a group setting [28]. In addition, as this type of approach often involves a small number of people, the technology developed may be overspecialized and relevant only to a few people [28]. Furthermore, involving users is not an easy task for researchers and designers in terms of time, energy, and competence. The added value of this type of approach (the benefits of using this approach vs the time and energy required) must be clear and explicit, which is not the case at the moment [13,28]. In addition, there may be disagreements within the group that lead to negotiations and compromises that result in inconclusive outcomes [19]. Moreover, stakeholders who are skeptical about applying this type of approach to digital technologies point out the difficulties for people with poor technological skills to contribute significantly to the design of the tool [15]. The well-known statement attributed to Henry Ford is relevant in this context: if the public had been consulted to improve the car, they would have added horses. At the individual level, people who engage in a participatory research process may experience physical and psychological exhaustion, stress, and financial loss [27]. Disappointment with the results of the process is also a possible outcome [27]. In the end, it is difficult to understand the mechanism underlying the beneficial effects of participatory research and co-design [29-31] and, more specifically, to determine the potential offered by co-design for the development of eHealth tools that would help reduce rather than exacerbate SHIs.

### Theoretical Framework

The development of eHealth tools with a view to reducing rather than exacerbating SHIs is a social justice issue. In this sense, the theoretical framework of social justice by Sen [32] and his capability approach shed light on the potential of co-design to reduce SHIs in eHealth at 2 levels: as a democratic process and as a conversion factor.

#### Co-Design as a Democratic Process

At a certain point, capabilities, defined as the real freedom that individuals have to *be* or *do* what they have reason to value [33], come to clash, namely, between individual and collective freedoms (or preferences). For example, the choice of having a free public health system, which theoretically allows everyone the freedom to take care of their health, implies a societal choice to contribute financially to support it. For Sen [32], only individual preferences that pass the democratic debate test deserve to be supported by public action insofar as this debate leaves as much space as possible for the individuals concerned [34]. Although Sen addresses this democratic process at the population level, his capability approach provides a relevant interpretive framework for the co-design process. Bonvin and Farvaque [34] explain that from Sen’s perspective, the democratic process has 3 values: (1) an intrinsic value that implies that the possibility of participating in a public debate is a fundamental capability, regardless of the final result; (2) an instrumental value where people can express their views and the latter are integrated into decision making; and (3) a constructive value that reveals that each way of considering a problem is a social construct and that it is important that the people concerned are involved in the process. This suggests the guidelines for the development of an eHealth tool in a democratic way: (1) supporting the participation of people at risk of SHIs in the co-design process, both through the opportunity to attend co-design sessions (CoDs) and through access to free speech and unfiltered information; (2) sharing decision-making power with people at risk of SHIs, including efforts to neutralize inequalities [34].

#### Co-Design as a Conversion Factor

Conversion factors are various personal, social, and environmental characteristics that positively or negatively affect an individual’s ability to convert their resources and formal rights into effective functioning [35]. In this study, conversion factors are facilitators and barriers to individuals’ use of eHealth tools to take care of their health. The participation of FUs appears to be a facilitator for the co-development of tools for people at risk of SHIs to the degree that it can make these tools more accessible and tailored to their needs.

### Objectives of This Paper

The context of this paper is a study aimed at exploring 7 conversion factors that must be considered in the development of eHealth tools, with a view to reducing SHIs. This paper focuses specifically on one of these factors, the participation of people at risk of SHIs in the co-design of an eHealth tool. The objective of this paper is to explore the ways in which co-design can support the development of an inclusive eHealth tool for caregivers of functionally dependent older persons. This paper is the third in a series of papers included in a thesis on the reduction of SHIs in an eHealth context [7,36]. The methodology behind this thesis is detailed in the second paper of this series [36] and is briefly summarized here.

## Methods

### Study Design

This study is part of a larger project titled, “Better meeting the needs of caregivers in providing safe home care for the functionally impaired older persons,” that the research team informally refers to as “the QADA project” in recognition of the fact that it is funded by the Ministry of Families as part of the Québec Ami des Aînés (QADA; Age-Friendly Quebec Program). The project is led by a group of researchers whose intent is to include the social justice perspective in their project (more details are given in the protocol of this project) [37]. The purpose of the QADA project is to develop an eHealth tool to facilitate the process of help-seeking for the caregiver of functionally dependent older persons. The QADA project is based on a co-design approach and thus allows us to achieve our objective.

This study is qualitative in nature, with what can be described as a social justice design since the concept of social justice, based on the capabilities approach, is involved in all phases of the study [38]. Using a qualitative approach was essential to describing the mechanism that potentially underlies the effect of co-design on SHIs [39].

### Population, Participants, and Selection Criteria

All QADA project co-designers are participants in this study. They fall into 4 categories: caregivers, community workers, health and social service professionals (HSSPs), and research team.

#### Caregivers of Functionally Dependent Older Persons

This population is at risk of SHIs because of they are much more at risk of developing health problems (physical and psychological) than the general population [40-42]. For the purpose of this project, any person who provides unpaid assistance on a regular (weekly) basis to a functionally dependent older person is considered a caregiver.

#### Community Workers and Health and Social Service Professionals

Given their proximity to caregivers, the possibility of obtaining an additional perspective and the desire to develop a tool that is complementary to what exists already, the choice to involve community workers and HSSPs as co-designers was relevant to the QADA project. The condition for participating was that they offer services or interact directly with caregivers of functionally dependent older persons (eg, nurses, home care providers, social workers).

#### Research Team

The members of the research team are the participants, and this is of key importance in this study, insofar as the integration of conversion factors must rest on an epistemological and methodological choice made by researchers and designers that must be applied in a realistic and concrete way. Their point of view, which will be largely experiential within the QADA project, is therefore crucial for the implementation of the recommendations resulting from this study. The QADA project research team initially consisted of 8 coresearchers. The participation of these individuals varies according to their availability and expertise. The members of the research team are the 4 participants involved in all phases of the project, and they included the QADA project director, an anthropologist and professional researcher, a user experience designer, and the author of this paper—a doctoral candidate in educational technology. The author of this paper was involved in this study as a participant observer [43], that is, the author took part in the preparation of the CoDs by ensuring the participation of FUs, facilitating the CoDs, debriefing CoDs, and developing the prototype from the results of the CoDs. However, the author played the role of observer when listening to recordings and analysis, steps that began once the co-design phase was completed.

### Recruitment

Recruitment was performed through the QADA project with a purposive sampling strategy [37]. The home care and support for the autonomy of seniors programs older adult care management of the 11 Integrated Health and Social Service Centres were contacted to recruit HSSPs. Members of community organizations were contacted directly via phone or email. They were asked to publicize our recruitment announcement among caregivers attending their institutions and activities. Finally, recruitment announcements for caregivers were posted to 30 family medicine groups throughout the province. The latter method of recruitment did not work. Caregivers were therefore recruited through the services of HSSPs and community workers, which implies that they were already service users.

### The Research Sites

The study took place in 11 Quebec regions (Côte-Nord, Mauricie, Centre-du-Québec, Capitale-Nationale, Chaudière-Appalaches, Montérégie, Bas St-Laurent, Gaspésie, Outaouais, Montreal, and Laval). The location of CoDs varied, depending on availability (eg, municipal or community premises or those connected with the HSSP network). The research team’s work sessions (preparation sessions) were held at the research center, sometimes in person, sometimes using Skype (Microsoft)—with members in remote locations. Table 1 shows the number and type of co-designers who took part in each CoD. Co-designers were invited to participate in the CoD held in their region. This meant that the co-designers were not the same from one session to another except for the 3 advisory committee (AC) meetings, where it was hoped that the participants would be the same.

**Table 1:** Number of preparation sessions required for the research team, the number and type of co-designers at each co-design sessions, and the content covered in co-design sessions.

CoD^a^	Number of preparation sessions required (n=24) by the research team^b^	Number and type of co-designers (N=74+4 research team members)	Content covered in co-design or AC^c^ sessions
CoD1	2	2 CWs^d^, 2 HSSPs^e^, 3 caregivers	Identification of caregivers’ needs
CoD2	1	1 CW, 1 HSSP, 4 caregivers	Identification of caregivers’ needs
AC1	1	2 CWs, 2 HSSPs, 1 caregiver	Final choice of needs and recommendations for the continuation of co-design
CoD3	1	2 CWs, 2 HSSPs, 2 caregivers	Exploration of existing functionalities that meet needs and identifying gaps
CoD4	2	2 CWs, 2 HSSPs, 1 caregiver	Brainstorming on the functionalities that can address the gaps
CoD5	3	3 CWs, 2 HSSPs, 3 caregivers	Choice of functionalities to be integrated into the tool and development of the site architecture
AC2	1	4 CWs (including 1 who also participated in AC1), 2 HSSPs (both of whom also participated in the AC1), 2 caregivers (including 1 who also participated in AC1)	Choice of functionalities that were not consensual
CoD6	3	4 CWs, 3 HSSPs, 3 caregivers	Functionalities and content development
CoD7	5	3 CWs, 2 HSSPs, 5 caregivers	Functionalities and content development
CoD8	3	4 CWs, 2 HSSPs, 7 caregivers	Functionalities, content development, and pretesting
AC3	2	4 CWs (including 1 who also participated in AC1), 2 HSSPs (both of whom also participated in the AC1), 2 caregivers (including 1 who also participated in AC1)	Exploration of the prototype, choice of realistic functionalities, and discussion on the content

^a^CoD: co-design sessions.

^b^The number of preparation sessions was not defined in advance but rather on an as-needed basis, depending on the evolution of the prototype and the complexity of the results analysis.

^c^AC: advisory committee.

^d^CW: community workers.

^e^HSSP: health and social service professional.

### Data Collection

Several stages of the QADA project focused on exploring the ways in which co-design can support the development of inclusive eHealth tools that contribute to reducing SHIs:

1. Preparatory meetings for the CoDs (including the AC) by the research team (n=24). These provided information regarding the efforts made to ensure the optimal mobilization of participants, obtain consensual decision making, and choose the information to be presented. The resulting documents (CoD planning) and the audio recording of these meetings were used as raw data for analysis.
2. CoDs (n=8 CoDs and 3 working sessions of the AC). These sessions produced information about the co-design process. The sociodemographic data of the participants (provided by them) and the audio recordings of these meetings served as raw data for the analysis.
3. Co-design postsession debriefing meetings (n=11). These meetings helped to quickly identify researchers’ perceptions of the co-design process. Note taking during debriefing and audio recordings also served as raw data for analysis. These meetings took place immediately after each CoD.

### Data Analysis

The analysis plan followed the method proposed by Miles and Huberman [44,45]. In this study, this resulted in a written summary of each document and audio recording from preparations of CoDs, the CoDs themselves, and debriefings. Deductive coding was performed to link the content relating to each conversion factor, including the co-design process, by using the MAXQDA software (Verbi) [46]. To refine the subthemes for the co-design process and prioritize the most relevant outcomes, the arguments for and against using a co-design approach in the development of an eHealth tool were transformed into analytical questions and used as a basis for presenting the results [47]. Textbox 1 presents these analytical questions. The same method was used to apply the democratic process guiding principles drawn from Bonvin and Farvaque [34] on the results (Textbox 2).

**Analytical questions based on arguments for and against using a co-design approach to develop an eHealth tool and the associated results.**

- Can co-designers significantly contribute to the development of an eHealth tool?
  Are they able to express their needs and preferences?
  Are they able to contribute even when they lack technological skills?
  Does their contribution lead to innovation?
  Can co-developers provide key information with a potential rapidly improve the effectiveness of the eHealth tool?
- Does the co-design approach involve only a small number of participants, which consequently makes the technology developed overspecialized and relevant only for a few people?
- Does co-design provide a reciprocal relationship between the research team and the people involved in the project?
  Does the FU's involvement increase the cultural and logistical relevance of the action in the community, support empowerment, and promote knowledge transfer?
  Do people who engage in co-design processes perceive beneficial effects on their physical and psychological health, self-confidence, self-esteem, empowerment, and social relationships or, on the contrary, physical and psychological exhaustion, stress, and financial loss?
  Do people experience disappointment with the results of the process?
- Do disagreements within the group give rise to negotiations and compromises that lead to inconclusive results?
- Is involving the FUs an easy task to accomplish for researchers and designers in terms of time, energy, and competence and is the added value of this type of approach clear and explicit?

**Analytical questions based on guidelines proposed by Bonvin and Farvaque for the development of an eHealth tool in a democratic way.**

- Have people at risk of SHIs had space to express themselves?
- Has the decision-making power been shared with people at risk of SHIs?

### Ethical Considerations

This project was approved by the Comité d’éthique de la recherche des Centres de santé et de services sociaux de la Vieille-Capitale (Research Ethics Committee of the Health and Social Service Centers of the Old Capital).

## Results

### Presentation of Results

The results are first presented in response to the arguments for and against using a co-design approach in the development of an eHealth tool. We then proceeded to lay out the manner in which we applied the democratic process, as described by Sen in our experiment. Finally, these results are discussed in relation to the research question, that is, in what way can co-design support the development of inclusive eHealth tools and consequently contribute to the reduction of SHIs. We begin by presenting the co-designer’s sociodemographic characteristics.

### Co-Designers’ Characteristics

A total of 78 co-designers participated in 11 CoDs, 24 preparation sessions, and 11 debriefing sessions. Table 2 presents the characteristics of the people who contributed to this research.

**Table 2:** Description of co-designers (N=78).

Sociodemographic characteristics		Caregivers (n=30)	Community workers (n=26)	Health professionals (n=18)	Research team (n=4)
**Sex, n (%)**					
	Female	26 (87)	20 (77)	18 (100)	4 (100)
	Male	4 (13)	(23)	0 (0)	0 (0)
**Age (years)**					
	Range	42-88	24-66	29-53	33-45
	Mean (SD)	77.9 (11.0)	44.8 (12.3)	39.6 (7.9)	40.7 (5.4)
**Education level, n (%)**					
	Elementary school	1 (3)	0 (0)	0 (0)	0 (0)
	High school	10 (33)	1 (4)	0 (0)	0 (0)
	College	4 (13)	4 (15)	6 (33)	0 (0)
	Vocational studies	1 (3)	0 (0)	3 (17)	0 (0)
	University	12 (40)	21 (81)	9 (50)	4 (100)
	None	1 (3)	0 (0)	0 (0)	0 (0)
	N/M^a^	1 (3)	0 (0)	0 (0)	0 (0)
**Age of the person cared for (years)**					
	Range	61-96	N/A^b^	N/A	N/A
	Mean (SD)	78.2 (9.9)	N/A	N/A	N/A
**Relationship with the person cared for, n**					
	Children	8	N/A	N/A	N/A
	Sibling	3	N/A	N/A	N/A
	Spouse	17	N/A	N/A	N/A
	Friend	2	N/A	N/A	N/A

^a^N/M: not mentioned by the co-designers.

^b^N/A: not applicable.

### Can Co-Designers Contribute Significantly to the Development of an eHealth Tool?

The majority of co-designers made a significant contribution at some stage of the prototype development. One co-designer even jokingly requested copyright:

Can we copyright this? (laughing).

Community worker CoD5

#### Were They Able to Express Their Needs and Preferences?

All co-designers (caregivers, HSSPs, and community workers) except 2 caregivers identified the priority needs (19 needs), analyzed eHealth tools to identify the desirable and missing features, created functionalities to address the unmet needs (25 functionalities), and created the website’s architecture (3 prototypes of low fidelity) and content (text written on the website, a questionnaire for the caregiver to identify their needs, a glossary to associate resource data, keywords that can potentially be used by caregivers in a search tool, and scenarios for potential videos). Some co-designers had a very clear vision of what the tool might look like:

A dropdown menu on the homepage. They will click on “issue affecting the older person” (Alzheimer’s disease) and they’ll find all the services available to the senior and the caregiver. They will recognize themselves based on the profile of the person to whom they provide care and through the search that they do for the senior. Menu based on the entry issue. There would be two entries, one “I am a caregiver” and the other “the person I provide care to has …. By diagnosis (for example, Alzheimer’s disease) or by issue facing the older person (for example, forgets to take their medication). Both! Both possibilities.

Community worker CoD2

#### Were Co-Designers Who Were Lacking Technological Skills Able to Contribute?

Although the majority of co-designers contributed to the development of the prototype, 2 caregivers were unable to participate in the design activity. Despite the efforts of the research team members to clarify the objectives of the working session, these 2 individuals did not seem to be able to make the link between their experiences and the objectives of the session. The team members understood that what these caregivers needed most was to talk and they lent an attentive ear to meet their needs:

What do you think of this website?

Member of the research team (CoD3)

At my house, the shower is downstairs and he (spouse) can’t use it. I bought armrests for the toilet.

Caregiver (watching an image of a bathroom with special needs equipment proposed by the website)

Ms. X didn’t know why she was there. KL repeated several times that she could leave and made sure that they had her informed consent. It was hard for her.

Debriefing, CoD3

In addition, it seemed difficult for some co-designers (both caregivers, HSSPs, and community workers) to think in numerical terms, especially at the beginning of the process, during brainstorming activities to create functionalities targeted to meeting needs (CoD1 to CoD4). On several occasions, the solutions or reflection focused on the health system and current services rather than on the use of digital technology to meet the caregiver’s needs. The research team repeatedly refocused the co-designers on the objective of developing a digital tool targeted to meet the needs of caregivers:

The system should coordinate their services with those of community organizations, including references.

Community worker, CoD1

Each CLSC should have a form where individuals can indicate their needs and streamline efforts where they are most needed. They could adapt services to needs.

Caregiver, CoD2

Technology is limited. TV advertisement worked really well. In Saguenay, the newspaper works well.

Community worker, CoD3

Financial compensation for meeting with the caregiver and conducting an assessment.

HSSP, CoD4

A follow-up by telephone with caregivers at least once per year.

Caregiver, CoD4

Social workers also used to think in terms of social services and it took some reframing in order to think digital.

Debriefing, CoD4

However, all HSSPs, community workers, and 28 caregivers contributed in one way or another to the development of the prototype.

An important finding was that caregivers who were less comfortable with technology and less talkative because of their difficulty in contributing directly to the designing of the tool explicitly agreed to take on the role of guardians of the literacy level:

Caregiver (CoD6): I don’t work hard. I’m a caregiver. I see how hard you work in organizations.

Community worker: we work for you.

Member of the research team: However, you play an important role in the group by saying “I don’t understand this word” or “What does this mean?” Your contribution is essential in this respect.

As a result, this caregiver went from being silent to regularly participating in her subgroup:

When you say home transportation, you mean that someone comes to our home to replace us? (…) Support group: what do you mean? (…) What is GMF?

Caregiver, CoD6

This contribution of caregivers both in speaking out and in ensuring that the tool respects the eHealth literacy level of FUs was then reinvested in the following CoDs.

#### Did Co-Designers’ Contribution Lead to Innovation?

The members of the AC raised the paradox of having, on one hand, an essential need to move beyond the usual reference frameworks to create an innovative eHealth tool and the challenge, on the other hand, of effectively moving beyond these reference frameworks in the search for solutions and creation activities, despite an awareness of this need:

We all come up with the same idea, i.e. a website offering information. We’re having a hard time thinking outside the box. We stick to the tried and tested.

Researcher, AC1

We shouldn’t repeat what we know already, it will make no difference.

HSSP, AC1

Websites, apps, video games, video clips, surveys, quizzes, writing letters. I personally am not able to think creatively.

Researcher, AC1

#### Were Co-Developers Able to Provide Key Information to Rapidly Increase the Effectiveness of eHealth Tool?

At different times and at different levels, the co-designers brought up key information that the team would not have thought to incorporate on its own. For example, the idea that the location of community resources must be presented by district, the importance of obtaining results with no more than 2 clicks, the fact that resources must be both self-regulated and solicited, and the fact that caregivers sometimes have to do research for several Quebec regions or for several profiles because they provide care to more than one person:

For Montreal, we’re facing the challenge of postal codes, CSSS territory: most organizations use the old CSSS system, but some are neighbourhood-focused. Searching by distance can’t work for Montreal because an organization may be near your home, but you may not have access to it because you’re covered by one that is 20 km away. Distance doesn’t mean anything.

Community worker, CoD6

I just want to browse fast, I don’t have time. I don’t have the time to sign up.

Caregiver, CoD5

Community worker, CoD6: Will the tool cover public and private service organizations?

Member of the research team: people will sign up with user verification. Community stakeholder: your data bank will be half full. We must also solicit resources.

The caregiver thought that she had to create a profile for St-Hyacinthe and for Drummondville because she provides care to two individuals.

Member of the research team, debriefing CoD8

### Does the Co-Design Approach Involve Only a Small Number of People, Which Consequently Makes the Technology Developed Overspecialized and Relevant Only for a Few People?

The co-designers, except for the research team, are potential FUs of the tool developed. The particularity of the methodology used (ie, having a different group of co-designers at each CoD) enabled the contribution of a greater number of co-designers to the project. As Table 1 shows, 74 of them were divided into 3 categories: caregivers, community workers, and HSSPs. Owing to their different roles, these 3 types of FUs had different experiences (and perspectives) in relation to caregivers’ help-seeking process. The sociodemographic characteristics of the co-designers highlight a variety of educational levels (primary to university level) and age range (24-88 years). However, there is a predominance of women. It appears that the co-design approach can be used with the involvement of a greater number of participants but, more importantly, with a greater diversity of participants to limit the risk of creating an overspecialized eHealth tool. However, this method has a set of challenges. Indeed, the co-designers had to agree to build the tool based on previous decisions that were not their own. The research team had to take about 30 min at each CoD to explain the progress of the project and the decisions made by the previous groups. In addition, this method did not allow the co-designers to develop their co-creative skills over the long term and limits the possibility of developing a long-term relationship of trust.

### Does Co-Design Provide a Reciprocal Relationship Between the Research Team and the People Involved in the Project?

#### Does the FU's Involvement Increase the Cultural and Logistical Relevance of the Action in the Community, Support Empowerment, and Promote Knowledge Transfer?

The research team and practitioners gained knowledge about the caregiver’s help-seeking process and the reality and issues facing community workers and HSSPs. The latter also discovered several other digital tools on caregiving, took advantage of the expertise of caregivers in a context other than the supportive-based relationship, shared advice with each other, and discovered existing services in regions other than their own:

It’s already quarter to three. Time flies. I like this, I learn things.

HSSP, CoD5

This is the first time I’ve participated in training as quick and well done as this. It’s not a waste of time.

Community worker, CoD1

The participation of caregivers made it possible to adapt the tool’s functionalities to their help-seeking process. This contribution was a unique learning experience for the research team, HSSPs, and community workers because caregivers described how they searched for help on the internet, especially in the physical and psychological state they find themselves in when they engage in help-seeking. This influenced, among other things, the development of the prototype in terms of functionality (as few features as possible so that the desired information would be found with no more than two clicks), the home page (search box highlighted on the home page), quick access to a contact person (phone number), the incentives for initiating contact with formal resources (eg, caregiver testimonial video and virtual tour of the site), and the importance of exchanges with other caregivers to initiate the help-seeking process:

The forum is of no help to me as a caregiver. Because if I post something, this makes one more page I have to check. I prefer talking with someone right away. I need contact, quickly. No time for the forum or the chat. Things are difficult today, I want to talk to a real person.

Caregiver, CoD5

I did a search on the X website. But sometimes it takes too long. We do it in the evening, we're already tired, it’s too taxing. I think the project must ensure that we can get to the information rapidly and that we can take rapid action.

Caregiver, CoD7

#### Did the Participants in the Co-Design Process Perceive Any Beneficial Effects on Their Physical and Psychological Health, Self-Confidence, Self-Esteem, Empowerment, and Social Relationships or, on the Contrary, Physical and Psychological Exhaustion, Stress, and Financial Loss?

The research team set up activities that were intended to be effective and fun. They wanted to create a friendly environment where everyone was free to drink, eat, and move around as they pleased. The objective was to create the prototype in a serene and safe environment. The recordings reveal that co-designers laughed together on several occasions.

Be very comfortable to move around at any time. We want it to be relaxed.

Research team, CoD6

Caregivers, for their part, discovered new services that exist in their region and shared their experiences with other caregivers, and some felt they were contributing for the benefit of caregivers in the future:

I think that this is promising for future caregivers. It’s a way to give back. Yes, they’ll be better informed, more knowledgeable, and more thanks to the project. It will be easier for them and better for the person receiving care.

Caregiver, CoD2

On the other hand, 4 caregivers and 1 HSSP verbally expressed emotions of anger, sadness, guilt, and fatigue unrelated to their participation in the project but related to their work or role. The team adjusted the schedule to allow for time to express these emotions. In addition, the team ensured that each time, the person had the necessary support following the CoD (often with the community worker or HSSP who was already on the site):

Resources are lacking. (...) There’s a lack of personnel; I wasn’t able to find anyone to cover nights. I slept 3-4 hours per night (crying). I slept in the basement. Human resources, self-employed workers, are lacking. We have to take this into consideration. Even with the best tools, if there is no one to provide the service, we’re running into a wall. The population is aging. The money in the healthcare system is poorly distributed.

Caregiver, CoD7

#### Do People Experience Disappointment With the Results of the Process?

At the time of writing, the co-designers had not yet seen the final result, with the exception of the members of the AC. The latter did not explicitly express satisfaction or disappointment with the prototype.

### Did Disagreements Within the Group Give Rise to Negotiations and Compromises That Led to Inconclusive Results?

Three major themes were the subject of dissension among the co-designers. The first concerns the difference between the needs identified in CoD1 and CoD2. The AC1 working session made it possible to decide to keep all the needs identified in the 2 CoDs:

What is the point in eliminating needs? Do we have to have a limit?

Researcher (AC1)

We can miss out on something.

HSSP

If we get rid of some needs and it turns out that the tool doesn’t meet a given need, maybe it would have met another. We don’t know. We might eliminate needs that are easy to respond to.

Researcher

I would keep them all and some can probably be grouped together.

Community worker

The second concerns the desire of caregivers to have a space for discussion with other caregivers, whereas community workers and HSSPs feared that this would open the door to people who are ill-intentioned and who would take advantage of the caregiver’s moment of vulnerability or offer the caregiver misguided advice. This discussion started in CoD3. This dissension was discussed at AC2 without finding a solution. However, the committee mandated the research team to find a solution that was acceptable to all. The dilemma continued until CoD7, where the co-designers proposed a solution that met the AC’s expectations, that is, to give organizations the opportunity to indicate on the website that they do twinning between caregivers (the twinning is therefore supervised by a professional).

We have to meet with people before they get too tired; right now, contacts are made while people are exhausted. If platforms for discussion are available, initial contact can be made and when things aren’t going so well, I know already that I have things in common with someone. (...)

Caregiver (CoD7)

I think matchmaking should be organized in regions where the need is felt and that local organizations should support it. This could be a service offered by these organizations. In their service offer they should put “matchmaking service to encourage twinning with a caregiver” and there, the caregiver can contact the organization to use the service.

Community worker

The third dissension concerned offering a system, on the website, for evaluating the services offered by organizations (similar to hotel and restaurant ratings). Organizations mentioned that such reviews are subjective and could affect their funding. Caregivers also pointed out that the work environment in their home is not always conducive to doing a good job and that it was not necessarily the worker’s fault. There is also a major difference between the service and the person providing the service. The caregiver may not be happy with a person who nevertheless provided high-quality service. It was a community worker who found a consensus solution:

It’s true that we don’t always dare to use services. If I leave and someone else is to provide care to the person that I love more than anything in the world, this caregiver enters my privacy, and this is disturbing. Do they have a criminal record, all employees (should be) screened? Providing elements of trust to caregivers.

D7

As a result, a number of statements that organizations can check if they apply to them were included. They appear in the caregiver’s search results. For example, all employees and volunteers have received training on neurocognitive disorders or the criminal record of all employees and volunteers is checked.

### Is Involving the FUs an Easy Task to Accomplish for Researchers and Designers in Terms of Time, Energy, and Competence, and Is the Added Value of This Type of Approach Clear and Explicit?

This question will be addressed in the *Discussion* section.

### Co-Design as a Democratic but Imperfect Process

#### Have People at Risk of SHIs Had the Space to Express Themselves?

The team sought to ensure that speaking time was shared during the CoDs, in particular by involving a number of facilitators. In addition, at the beginning of each session, clear instructions were issued to encourage equality of expression:

Give everyone a chance to speak; those who tend to take a lot of space are asked to give others a chance, and those who are reluctant to speak are invited to make an effort to participate. We would like to hear what each of you has to say.

Team member, CoD1

In addition, the facilitator tried to reach out to less-talkative participants:

I see you nodding (to a person who didn’t speak much).

Team member, CoD2

However, it was also important to respect people’s personalities:

I asked people who don’t speak up if they have something to add in response to what was just said. At the same time, it’s putting them on the spot. Not easy. We want people to talk, but we don’t want to force them.

Debriefing, CoD4

Other measures were put in place to support discussion, including limiting CoD groups to 6 to 12 people and introducing subgroup activities with 2 to 3 participants and a facilitator:

In small groups, everyone spoke, unlike in the plenary session where one lady did not speak at all. People liked this.

Debriefing, CoD3

In particular, the team anticipated that community workers and HSSPs would take up a lot of space, unlike caregivers. Initially, the facilitation plans called for the use of small groups with a facilitator and dividing participants according to their roles (eg, community workers together, HSSPs together, and caregivers together). In fact, the team found an equivalent level of input in the first CoD. However, the cross-fertilization of expertise within a subgroup also appeared to generate more creativity:

For the next co-design session, I‘d mix up participants. We have to foster creativity and for this reason, it’s good to mix participants to stimulate the flow of energy. We want divergent thinking.

Preparation, CoD4

In addition, in mixed subgroups, some HSSPs and community workers sometimes helped caregivers to speak out:

In the definition, the social worker got the caregivers to speak. She asked them “What made you realize that you were caregivers?” They brought up important notions, key elements.

Debriefing CoD6

However, this was not done always:

Team member to a caregiver CoD6: Does this sound familiar to you?

Caregiver: yes... (tried to answer, but was interrupted by an HSSP).

The team’s observation was that speaking out depended on personalities more than the role or status of each person (a caregiver, an HSSP, or a community worker), and it was therefore difficult to plan in advance:

Are we going to fail to get the caregivers’ opinion because healthcare and community workers will be speaking at the time? We can’t anticipate this.

Preparation, CoD2

In short, the objective of the workshop, the need to combine expertise or absence thereof, and the personality of co-designers were all factors that influenced the relevance of using mixed or homogeneous subgroups. For this experiment, the latter failed to guarantee shared speaking time. The presence of a facilitator in small groups was more effective in ensuring equal opportunity for expression.

#### Was the Decision-Making Power Shared With People at Risk of SHIs: A Shift From Ideal to Pragmatic Considerations

Initially, the team proceeded with a concern for making participants the main decision makers in the development of the tool. It aimed to be as neutral as possible so as not to influence participants:

Caregiver CoD5: It depends on your needs (speaking of the website).

Team member: We don’t have specific needs. It’s up to you to decide.

(Speaking of co-designers) They are the ones creating it (the tool).

Preparation, CoD2

However, it must be admitted that several barriers led to the team becoming increasingly involved in decision making. First, the team indirectly influenced the CoDs by preparing facilitation and providing materials to support the participation of the co-designers:

We’ll start with a list of needs drawn from the literature and the pilot project. But we need to reformulate these needs in order to facilitate group discussion. Ideally, the reformulation would be done by the group but because we’re short on time, the research team will do this (clean up) and provide the group with a list. Therefore, the research team necessarily filters information. However, the group will have the possibility to take out, add, or reformulate things.

Preparation, CoD1

Second, by condensing data from the sessions required to analyze the data to plan the next meeting:

The next step is juxtaposing results. Each subgroup will transcribe this, and we’ll look at this at the preparatory meeting Thursday morning.

Debriefing, CoD6

Analyzing the results is another factor. Although the research team had the rigor to cross-reference the analyses, this cross-reference was carried out by the research team:

Everyone will have to review X’s analysis to confirm that it corresponds to what we’ve addressed.

Preparation, CoD4

The AC’s initial role was to be the final decision maker in cases where the groups had divergent opinions or if proposals appeared unrealistic. It also had the responsibility to ensure that the decisions made by the co-designers in the development of the prototype were respected. However, the AC was not an operational committee and, in this sense, it took place only 3 times in the process:

The advisory committee meeting is far, choices will probably have to be made and we’re the ones who will make them in the end… (to prepare the next co-design session).

Preparation, CoD4

Moreover, there can be divergent opinions within the AC itself. Among other things, caregivers wanted to have the possibility to be matched with other caregivers; however, HSSPs and community workers feared that this would expose them to ill-intentioned people. This difference of opinion was brought to the AC, which was incapable of deciding. The research team proposed changing the weighting of opinions in favor of the caregivers. However, the AC mandated the research team to mediate a solution. The team brought the debate back to the CoDs twice and arrived at a solution.

Finally, the co-design approach requires a substantial amount of time. Time constraints (the project duration and the financial support to assume the number of CoDs) led the team to make decisions in view of subsequent sessions and move the prototype forward. This constraint was anticipated from the outset:

From a practical point of view, decisions regarding data will probably be taken by the research team. Although we’d like all decisions to be made in the co-design context, we must recognize that time (and the fast pace of the project) will force us to make decisions outside of co-design.

Preparation, CoD1

Team member 1: Yes, we are pressed for time right now. I don’t feel comfortable. We’re making plenty of decisions. [Preparation CoD7]

Team member 2:You’re right, we’re moving away from co-design.

Team member 3: We want to push because there is a deliverable. I’m getting more and more uncomfortable. We will have no choice but to make a lot of decisions. [Discussion between team members, Preparation CoD7]

Once the team realized that, from a pragmatic standpoint, not everything could be done within the scope of CoDs, it had to set apart topics that had to be discussed in sessions and those that were less impactful on the overall quality of the co-design process, which it could decide on itself.

Issues pertaining to the methodology were left to the research team. Some features deemed unrealistic in this study were also removed by team members. The importance of the final decision and the availability of information were also the factors that determined whether or not to discuss an issue in a CoD:

Team member 1: We’re in the process of making an important decision. [Preparation CoD7]

Team member 2: This aspect should be addressed in a co-design setting. This is important.

Team member 1: Being kept abreast of activities in my region…We wouldn’t have gone any further in our thinking. [Preparation CoD7]

Team member 2: Shall we take this to co-designing? (...)

Team member 1: We have no choice since we don’t know (...) We’ve never discussed this from this vantage point. To be moved to the co-design setting. [Preparation CoD7]

## Discussion

### Principal Findings

In this section, we will answer the research question in light of the aforementioned results, that is, how co-design can support the development of an inclusive eHealth tool for caregivers of functionally dependent older persons and consequently contribute to the reduction of SHIs.

The first question: Can people in situations of vulnerability (caregivers in this case) with no expertise in design contribute in a significant manner to the design of an eHealth tool and provide insight to help make the tool more inclusive?

In the Introduction section, we presented the 2 dominant schools of thought on this question. One posits that people can contribute meaningfully by expressing their interests, attitudes, beliefs, values, and expectations, by demonstrating their capacities [10,18], and by describing the context in which they will use the eHealth tool [25]. In addition, they can identify what is useful and relevant [21]. Their participation may also lead to innovation [24]. On the other hand, skeptics of the co-design approach timidly argue that people may not be aware of their needs, be able to express their needs, or want to discuss their needs in a group setting [28]; that the technology developed can be overspecialized and relevant only to a few [28]; and that people will have difficulty contributing significantly if they do not have technological skills [15]. Our position? Somewhere between these 2 paradigms, we advocate the use of co-design while keeping a critical eye. On the basis of our experiment, some caregivers did have skills and knowledge in digital technology, which accounts for the important role they played in the development of the prototype. Two caregivers were unable to contribute to the development of the prototype in any way. However, with the exception of these 2 individuals, all caregivers (including HSSPs and community workers) made a significant contribution to the process. Some reframing by the research team was necessary to ensure that the solutions found were linked to the digital tool rather than focused on the current health system and services. Nevertheless, most co-designers made a key contribution, some directly on the prototype by offering creative ideas and key information and others indirectly by ensuring consistency between the tool and an adequate level of literacy and by respecting the caregivers’ help-seeking process. If the use of a co-design approach, particularly with people at risk of SHIs (in this case caregivers), facilitates the integration of other conversion factors such as eHealth literacy and help-seeking, it therefore contributes to the development of universally accessible tools and potentially contributes to curbing SHIs. The co-design approach maximized this participation beyond what could have been expected by the research team, that is, key information was provided that could not have been anticipated by interview questions, for example. This tends to support the view that involving FUs in the development of a prototype allows for going beyond what can be seen in an observation or heard in an interview [16,17]. In this sense, the time and energy invested in the co-design yield added value.

However, let us take this reflection a step further and answer the following question: Can co-design be considered a democratic process, as described by Sen? Can an equal distribution of decision-making power be implemented between the different co-designers? In all humility, the answer to this question is more nuanced.

In this experiment, the role of the research team in co-designing and its neutrality in the research project were ambiguous. Its original intent was to remain neutral to leave the decision-making power entirely in the hands of the co-designers; however, its position evolved to where the team defined itself as a co-designer in its own right and, finally, to become a more important decision maker, all the while respecting the decisions made by the other co-designers. The underlying reflection varied over time and between researchers. It seemed impossible for the research team to not intervene directly in the development of the tool and therefore to make decisions without debate with the co-designers. What practical impact did this choice have on the democratic process, as understood by Sen?

Bonvin and Farvaque [34] invited us to question our interpretation of what limits or hinders democratic development and, consequently, of what can be an obstruction factor in the development of capabilities. They address the importance of people having access to debate, facilitating their expression, and including their views in the decision-making process. In this experiment, caregivers who wanted to contribute to the development of the eHealth tool were all welcomed regardless of their level of education, digital literacy, or technological skills. The research team put in place various means to ensure free discussion and the expression of different points of view. Participants’ values, including those related to the help-seeking process, were incorporated into the tool. Two decision-making power—in other words, democratic—issues (obstruction factors) arose. The first was a divergence of choices between caregivers and stakeholders (community and health network). It was decided to seek a solution acceptable to all parties through mediation. These tensions can be seen as democratic limitations resulting from the choice to involve stakeholders in the design process, but they can also be seen as creative resources that are part of the co-design method because they bring to light issues that would have emerged sooner or later [48].

The second was when the time allocated for the project ran out. At that point, some issues had to be left out of CoDs. The lack of time prompted the team to discern what was essential to designing an inclusive tool versus decisions that did not have a democratic stake. It would have been valuable (and even important) at that time if a caregiver could contribute to this reflection. However, the presence of a caregiver to help with the preparation meetings would have been impossible because of availability constraints. The team had 1 to 4 preparation meetings per CoD, often in less than a month. We anticipated the negative effects discussed by Attree et al [27]. It would be useful to single out some key issues to guide researchers on what should be addressed in co-design discussions and what can be left out to make optimal use of this approach in a real-world context. For example, is this decision consistent with all the information we have received so far in the context of the co-design process? Does this decision benefit some people to the detriment of others?

Looking at the spectrum of public participation in the International Association for Public Participation [49], it can be broadly said that, on the whole, stakeholders’ involvement was limited to having a collaborative role, that is, the research team sought the advice and innovative ideas of the FUs and, to the extent possible, took them into account in the decisions it made. Could it have been otherwise? Theoretically, a genuine sharing of power requires participants to have an enabling role. From a pragmatic standpoint, is this one of the limits of co-design, and even an epistemological limit?

Individual interviews with co-designers would complement this reflection. How did they experience the co-design process? How did they feel about the project? Did they feel they had the opportunity to express themselves freely? This is the subject of a doctoral thesis by one of the members of the research team (MT). Data analysis is currently underway.

### Strengths and Limitations

To ensure the credibility of this study, the data collection was spread over a period of one year and involved a variety of participants (caregivers, community workers, HSSPs, and coresearchers) having various profiles (age, comfort level with technology, etc). Triangulation of the data was done through the use of various sources of information, including recordings of the preparation sessions, co-design, debriefing sessions, artifacts produced, and notes taken during the work sessions. In addition, the accuracy of the summary documents was verified by a member of the research team (an anthropologist and research professional) who participated in the working sessions, co-designing, and debriefing. She checked the accuracy of 10% of the documents at random. This study is the subject of a thesis and is therefore supervised by a thesis committee composed of 4 university researchers in the fields of education and health. However, it also has limitations. Although we sought to include a variety of caregiver profiles, most co-designers were women and White. This may have had an effect on the interpretation of results concerning, among other things, the process of help-seeking, which is a cultural construct [50]. In addition, the majority of participating caregivers were retired and were already service users. Caregivers among the active population or caregivers at the beginning of the help-seeking process could have made different choices. Moreover, 12 of the 30 caregivers who participated had a university degree. According to Sen, only individual preferences that pass the democratic debate test deserve to be supported by public action insofar as this debate leaves as much space as possible for the individuals concerned. Although all caregivers are equally concerned about the tool as the others, in the context of fighting SHIs, the higher education of some may have influenced certain choices, including the content developed.

### Conclusions

The use of a co-design approach does not guarantee that participants at risk of SHIs will bring innovation, but it does guarantee, if their participation is promoted, that the tool developed will respect their process of help-seeking and their literacy level and potentially contribute to curbing the existing SHIs. Once the eHealth tool developed as part of this project is finalized, a usability study comparing people at risk of SHIs and people who are not at risk will confirm or invalidate this hypothesis. However, this approach requires time, and it is difficult to achieve the time and money constraints associated with research grants. Key issues supporting the reasoning of researchers and designers on what must be absolutely debated in the development of an inclusive eHealth tool could allow for optimal and realistic use of time.

## Abbreviations

AC: advisory committee
CoD: co-design session
FU: future user
HSSP: health and social service professional
QADA: Québec Ami des Aînés
SHI: social health inequality
